# Recent Advances in Automated Structure-Based De Novo Drug Design

**DOI:** 10.1021/acs.jcim.4c00247

**Published:** 2024-03-14

**Authors:** Yidan Tang, Rocco Moretti, Jens Meiler

**Affiliations:** † Department of Chemistry, Vanderbilt University, Nashville, Tennessee 37235, United States; ‡ Center for Structural Biology, Vanderbilt University, Nashville, Tennessee 37240, United States; § Institute of Drug Discovery, Faculty of Medicine, University of Leipzig, 04103 Leipzig, Germany

**Keywords:** Computer-aided drug design, Structure-based drug design, *De novo* drug design, Artificial intelligence, Machine learning, Genetic algorithm, Evolutionary algorithm, Fragment-based ligand design, Fragment growing, Synthetic accessibility

## Abstract

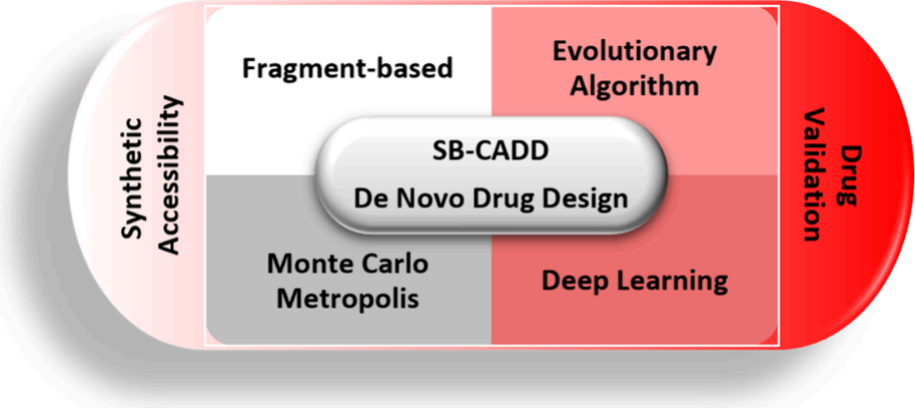

As the number of determined and predicted protein structures and the size of druglike ‘make-on-demand’ libraries soar, the time-consuming nature of structure-based computer-aided drug design calls for innovative computational algorithms. *De novo* drug design introduces *in silico* heuristics to accelerate searching in the vast chemical space. This review focuses on recent advances in structure-based *de novo* drug design, ranging from conventional fragment-based methods, evolutionary algorithms, and Metropolis Monte Carlo methods to deep generative models. Due to the historical limitation of *de novo* drug design generating readily available drug-like molecules, we highlight the synthetic accessibility efforts in each category and the benchmarking strategies taken to validate the proposed framework.

## 1 Introduction

Computer-aided drug design (CADD) methods have become more powerful as better hardware and novel methods like machine learning improve the performance of traditional tools.^1^ Structure-based (SB) methods such as docking and molecular dynamics play a crucial role in CADD, enhancing our understanding of how small molecules bind to the protein target.^2^ As more and more experimentally determined structures of therapeutic targets become available via X-ray crystallography, nuclear magnetic resonance (NMR) spectroscopy, or cryo-electron microscopy (cryo-EM), SB-CADD methods have sped up numerous drug discovery campaigns. The influence of SB-CADD methods expanded even more as homology modeling bridges the gap between similar protein sequences and determined structures.^3,4^ The recent success of AlphaFold in the 14th Critical Assessment of Protein Structure Prediction (CASP) showed the feasibility of highly accurate large-scale structure prediction, leading to the extensive AlphaFold Protein Structure Database filled with more than 200 million structures.^5,6^ Despite the improvement in computer hardware, the computational cost for evaluating a protein–ligand complex is still high, limiting the scope of assessment during hit searching. SB-CADD virtual screening campaigns can now screen ultralarge make-on-demand libraries containing millions of molecules, but this covers only a small proportion of the vast drug-like chemical space which is estimated to be up to 10^60^ molecules.^7^ For a search problem of this scale, exhaustive search is infeasible. The situation calls for more efficient ways of exploration.

*De novo* drug design refers to a subset of methods that aim to design novel molecules with pharmacological properties from scratch.^8^ Compared with SB-CADD virtual screening, *de novo* design can explore a wider chemical space in a time-efficient manner. Similar to SB-CADD virtual screening, the molecules proposed from *de novo* design are usually still far from a final drug, but they serve as good starting points for medicinal chemistry to develop. A *de novo* drug design workflow generally consists of candidate sampling and property evaluation, usually in an iterative fashion. Ligand property evaluation is generally performed through various scoring functions and pharmacological filters. The sampling method, or molecular construction, is usually the main difference between design approaches.^9^

**Figure 1:**
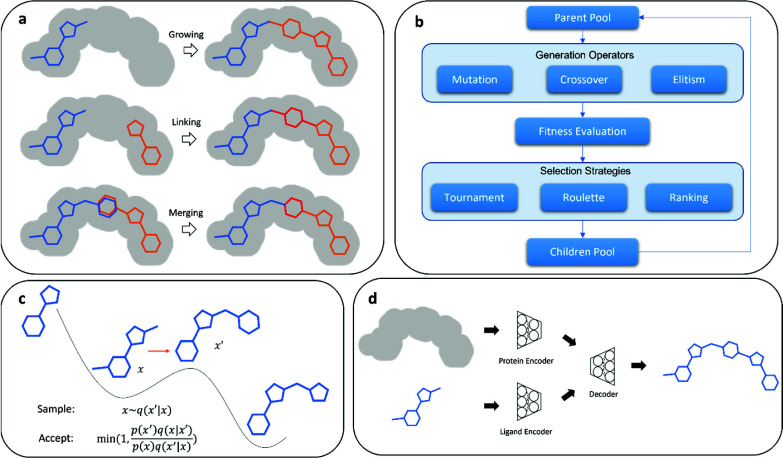
Schematic illustration of ligand sampling methods in structure-based de novo design. (a) Fragment-based growing, linking, and merging. (b) Evolutionary algorithms. (c) Monte Carlo Metropolis methods. (d) Deep generative neural networks.

Various sampling methods have evolved significantly over the past years. The first SB-CADD *de novo* design method, LEGEND (1991), employed an atom-based sampling method, placing atoms and bonds successively in the receptor pocket to explore the chemical space.^10^ However, the combinatorial explosion associated with atom-based methods soon drove the field toward fragment-based methods and computing heuristics. Conventional fragment-based sampling methods employ three major strategies: growing, linking, and merging to develop binding fragments into complete drug molecules.^11,12^ Evolutionary algorithms are a class of methods extensively used in *de novo* drug design. Mechanisms inspired by biological evolution are applied in these methods to optimize ligand population generation by generation.^13,14^ Monte Carlo Metropolis (MCM) is another sophisticated heuristic for sampling in high-dimensional space. Such methods have been applied in drug discovery to search step-by-step in the chemical space for drug candidates.^15^ Recent advancements in machine learning (ML) have brought numerous deep generative models into drug discovery, combining and redefining the tasks in a *de novo* drug design workflow.^16^ This review introduces recent sampling methods ranging from conventional fragment-based methods and evolutionary methods to emerging ML methods (Figure 1).

The scoring task in SB-CADD *de novo* design requires a balance between accuracy and computing time to accomplish iterative searching in a vast library within a reasonable time. Major scoring functions include physics-based force fields, empirical potentials, and knowledge-based scoring functions. Compared with quantum-level calculations, these scoring functions are not only less accurate but also more lightweight in computing resources. Newly developed scoring functions are regularly assessed by their scoring and ranking power, as well as docking and screening power, as in the comparative assessment of scoring functions benchmark.^17^ Besides conventional scoring functions, ML scoring functions have emerged over the years, though the scoring performance has been questioned to be dependent on training sets.^18^

While predicted binding affinity is the most commonly used evaluation strategy, other metrics including structural similarity to known binders, binding mode, validity, novelty, diversity, and drug-likeness are also frequently employed during or after the design. Another important property for evaluation is synthetic accessibility (SA). SA has been a consistent challenge for the field since its inception. Though a number of methods in the past decade have discovered chemical entities that eventually proceeded to experimental validation, manual alteration to proposed designs prior to actual synthesis is still a frequent occurrence.^19^ Many recent approaches have tried to resolve the SA issue, and their efforts will be discussed in this review.

This review will also examine how recent approaches have benchmarked and validated their methods. Although experimental validation is widely agreed to be more convincing than *in silico* evaluation, few protocols have validated their designs *in vitro* or *in vivo*, mostly because of SA concerns. Instead, *in silico* validation frequently relies on docking studies and molecular dynamics (MD) simulation. Methods differ in the selection of evaluation metrics for benchmarking, as well as their choice of protein targets. These differences will be outlined for each method to compare similar protocols in parallel.

## 2 Growing, Linking, and Merging

Fragment-based drug discovery usually begins with a screen of diverse fragments, often by computational virtual screening, but sometimes with *in vitro* methods. Compared with larger and more complex molecules that are less likely to bind, fragments bind less tightly but more reliably.^11,12^ These fragments are selected to form a library for further expansion into larger optimized molecules. Common fragment expansion strategies are growing, linking, and merging. Growing is the most commonly used strategy. Growing starts with a single core in the pocket, and subsequent additions of fragments then aim to extend the ligand into the rest of the pocket with improved affinity. Linking starts with two fragments occupying different nonoverlapping portions of the pocket, and the goal is to find a linker with suitable flexibility to maintain the original fragments’ binding modes. Merging combines the two fragments in different but overlapping parts of the pocket, with the common structure forming the core. Table 1 summarizes the program packages in the past eight years that adopted one or more of the fragment-based strategies.

**Table 1:** Recent Fragment-Based Ligand Design Packages

Method	Ligand Construction	Synthetic Accessibility	Validation (method; target)
**LigBuilder V3**(20)	Growing/Linking/Genetic Algorithm	Retrosynthesis analysis	(Prototype) *in vitro*; COX2/LTA_4_H
			(Full protocol) *in silico* MM/GBSA method; HIV-1 protease/reverse transcriptase
**NAOMInext**(21)	Growing	Reaction-rule based	*In silico* docking/alignment; aurora A kinase, carbonic anhydrase II, acetylcholine-binding protein, protease factor VIIa
**PINGUI**(22)	Merging	Reaction-rule based	*In vitro*/*In silico* docking; β_2_AR
*de novo***DOCK**(23)	Growing	Torsion environment from synthesizable database	*In silico* docking; HIVgp41
**AutoT&T 2**(24)	Merging	Real molecule reference library	*In silico*; angiotesin converting enzyme, VEGFR2, β-lactamase
**OpenGrowth**(25)	Growing	Fragment connection probability based on drug library	*In silico* MD; HIV-1 protease
			*In vitro/In vivo*; PDE3A-SLFN12 complex^26^
**Frag4Lead**(27)	Growing	Commercially available fragment database	*In vitro*/*In silico* docking; aspartyl protease endothiapepsin
**LeadOp+R**(28)	Growing	Reaction-rule based	*In silico* MD; Tie-2 kinase, human 5-lipoxygenase
**AutoCouple**(29)	Growing	Reaction-rule based	*In vitro*/*In silico* MD; CBP bromodomain

### 2.1 Ligand Construction

Fragment-based ligand construction usually begins with the selection and placement of anchors or growing centers. Most methods take a docked or cocrystallized ligand directly as the starting point^21,22,24,25,28^ or as the reference to lookup analog anchors from a given fragment library.^27,29^ LigBuilder V3 has a *de novo* design mode called Chemical Space Exploring Algorithm, which performs iterative growing and fragment extraction operations on a pool of seed structures derived from a single sp3 carbon, hence avoiding preassigned seed structures and allowing broader exploration in the chemical space.^20^*De novo* DOCK generates building block libraries including anchors from ZINC, breaking the molecules at each rotatable bond into rigid fragments, which are then oriented to the binding site via a graph matching algorithm.^23^ In some methods, the user also needs to specify the sites of optimization on an anchor.^28^ For most reaction-rule based methods, this step is unnecessary, since the reaction patterns automatically define the sites.

Building block sampling is a 2-fold problem: chemical space and conformational space sampling. Some fragment-based approaches therefore require docking of the fragment library to filter out undesired structures before ligand construction.^22,24^ Fragment sampling for reaction-rule based methods is straightforward since candidates are restricted to reagents compatible with the reaction. Methods that are not reaction-rule based derive fragment connection probabilities from real molecules and use these to guide ligand construction.^23,30^ AutoT&T2 limits the search space by searching for matched bonds between the reference library and the input lead molecule and carrying out a systematic crossover for all matches.^24^ Frag4Lead collects hit analogs from commercial databases, with the common substructure aligned to the input hit.^27^ Conformational sampling is typically through docking and in-site optimization of the product before the next iteration. NAOMInext generates conformations on the fly with a dynamic strategy that switches between breath-first-search and depth-first-search.^21^

Besides ligand flexibility, protein conformation is another factor to consider during design, though most methods only sample side-chain flexibility. OpenGrowth simultaneously grows ligands in several conformations of the protein, together with a rotamer search and geometry optimization on the chosen fragment, taking both protein and ligand flexibility into account.^25^ LigBuilder V3 allows multitarget design, targeting multiple binding sites or multiple conformations of a protein. Specifically, the multitarget growing mode synchronously grows identical fragments at the same growing site to maintain 2D structure consistency, while 3D conformations are independently optimized in corresponding targets. The ensemble linking mode grows each fragment independently and flexibly before attempting to link among suitable ones.^20^

### 2.2 Synthetic Accessibility

SA concerns in fragment-based methods usually arise at two stages: fragment source and fragment connection. The first is addressed through using a commercially available fragment library^27^ or, at the very least, having a real molecule/drug library as reference.^23−25^ The second is addressed in some approaches by ensuring connection validity with reaction rules and reaction-based fragment libraries.^21,22,28,29^ Fragment-based methods today are mostly iterative. The absence of SA supervision during fragment connection can lead to synthetically inaccessible molecules, especially at later iterations. Reaction-rule based approaches are therefore a general trend, particularly given the increasing number of reaction databases in recent years.^31^ Moving ahead, the concept of make-on-demand libraries presents promising prospects for the advancement of fragment-based methods. Enamine has generated a combinatorial library containing an impressive 36 billion readily accessible molecules through the enumeration of products from their existing compounds and reactions.^32^ This strategy significantly mitigates SA concerns at both the fragment source and fragment connection stages. These vast and dynamic libraries are anticipated to be instrumental in future fragment-based methods.

### 2.3 Validation

**Figure 2:**
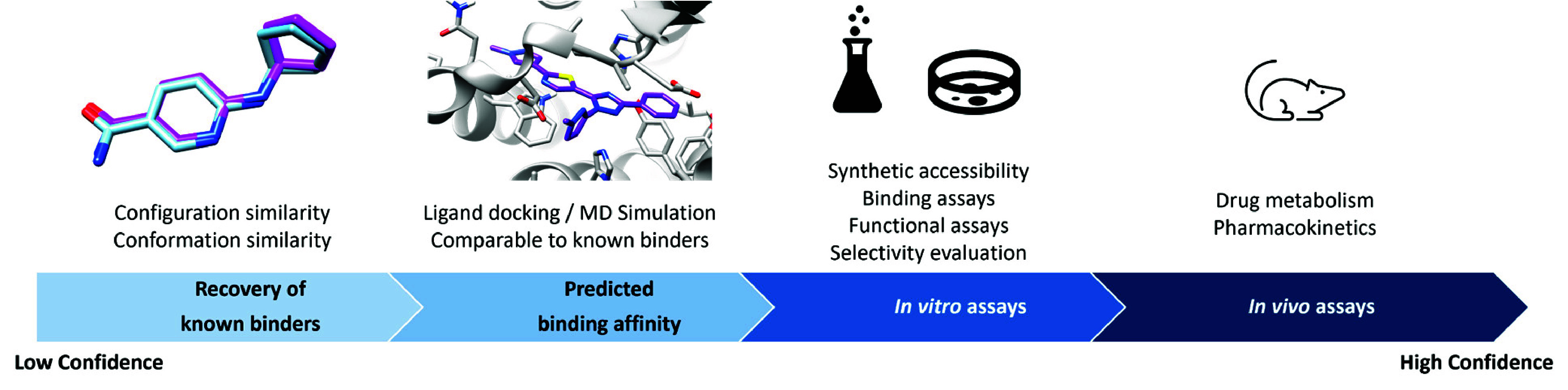
Levels of confidence for drug design validation.

Methods reviewed here were validated at different levels (Figure 2). At the very least, the methods were tested to see if the known binders or analogs could be reconstructed from a set of precursors or seed structures. The successfully recovered molecules from these methods also adopted poses within 2.0 Å root-mean-square deviation (RMSD) to native structures.^21,23^ However, such validation cannot test a method’s ability to generate novel molecules or to extract good ligands from a larger chemical space. Therefore, a majority of methods estimate the binding affinity of the compounds designed with generic databases, through docking or MD simulation. The reported methods mostly have top molecules estimated to be as potent or more potent than known binders or FDA-approved drugs.^20,25,28^

A few methods verified their designed ligands *in vitro*. A prototype of LigBuilder V3 has been experimentally validated by designing a COX2/LTA_4_H dual-functional inhibitor and yielded a ligand binding to COX2 and LTA_4_H with an IC_50_ of 7.1 and 7.0 μM, respectively.^33^ PINGUI found four ligands targeting the β_2_-adrenergic receptor (β_2_AR) with improved binding affinity over the core fragments used as growing centers.^22^ Frag4Lead proposed ten binders targeting aspartyl protease endothiapepsin that have been confirmed by X-ray crystallography, with the best-proposed molecule improving affinity over its reference by 266-fold and two compounds having affinities below 10 μM.^27^ Five molecules targeting CBP bromodomain and proposed by AutoCouple have been confirmed as nanomolar binders with the best one having a 10-fold increase in K_d_ compared with the known binder. The authors of AutoCouple then completed a series of optimizations to the proposed molecules to identify a hybrid molecule with good binding affinity and selectivity confirmed *in vivo*.^29^ OpenGrowth was applied to generate anagrelide analogs as potential molecular glues between phosphodiesterase 3A (PDE3A) and Schlafen 12 protein (SLFN12). The *in vitro* apoptosis induction activities of 14 synthesized analogs increased significantly with the best one having an IC_50_ of 0.3 nM. Further *in vivo* testing of the best compound also showed better tumor growth inhibition than the known drug anagrelide.^26^

For reaction-rule based methods, it is also important to confirm the validity or viability of the proposed synthetic routes. While molecules tested *in vitro* automatically prove their SA, methods lacking experimental validation have sought verification from the literature. For example, the synthetic routes suggested by LeadOp+R match past experimental studies in the literature, and nine of their proposed molecules for Tie-2 kinase were found to have been synthesized before.^28^ However, such validation cannot prove the SA of novel molecules that have not been synthesized.

## 3 Evolutionary Algorithms

Evolutionary algorithms (EAs) are powerful approaches for solving search and optimization problems that involve multiple, conflicting objectives. They mimic the concept of Darwinian evolution in that the fittest molecules are selected generation by generation. The genetic algorithm (GA) is the most commonly used type of EA.^34^ Other types include genetic programming, evolutionary strategy, and evolutionary programming.

An EA starts with an initial parent population (often randomly chosen). In the case of drug design, the initial population is usually a set of chemical compounds. Random, biological evolution-inspired operations such as reproduction, mutation, selection, and crossover are applied to individuals in the parent population to produce the “children”. Mutation introduces new information into the population, and crossover combines information from existing individuals to generate new populations. GA also employs the replication (or elitism) operator to carry the fittest molecules unchanged into the next generation. All “children” structures in the new population not only will be evaluated with a fitness function, usually in some form of the binding affinity, but can also involve properties such as drug-likeness, toxicity, and similarity to known actives.

Various selection strategies involving the “Roulette wheel”, “Tournament”, and “Ranking” are employed in each round to select a diverse set of fit molecules to function as the parent population in the next round. “Roulette” assigns an area weighted by fitness to each proposal on a metaphorical roulette. By giving each proposal a chance, roulette introduces randomness into each generation, and the exploration is less likely to be trapped in a local minimum. “Tournament” randomly samples a subgroup from the proposals and picks the fittest ones. “Ranking” directly chooses the best-scoring proposals but has the risk of selecting low diversity compounds at later generations with high convergence.^35^ While each strategy has its pros and cons, many EA methods incorporate more than one strategy to balance randomness, fitness, and diversity.

The iteration of offspring generation, evaluation, and selection continues until a user-set termination criterion is met, at which point the molecules will have converged to a set of locally optimized “fittest” compounds, substantially better than the initial pool. With independent runs of the EA starting from different sets of initial populations, the vast chemical space can be efficiently explored. EAs have been widely used in *de novo* drug design over the past two decades. Here, we summarize some recent EA methods to complement existing reviews^9,13,14^ on this topic (Table 2).

**Table 2:** Recent *De Novo* Drug Design Methods Using Evolutionary Algorithms

Method	Ligand Construction	Synthetic Accessibility	Validation (method; target)
**Dock_GA**(36)	GA	Torsion environment from synthesizable database	*In silico* docking; protein ligand complexes from SB2012 testset, SAR-CoV-2 M^pro^
**SECSE**(37)	Rule-based/GA	Retro-synthesis module	*In silico* docking; phosphoglycerate dehydrogenase
**EMGA**(38)	Evolutionary Strategy/Transformer ANN	SA scores	*In silico* MD; SAR-CoV-2 M^pro^
**Steinmann etal.**(39)	Graph-based GA	SA scores and filers	*In silico* docking; chorismate mutase, β_2_AR, DDR1, β-cyclodextrin
			*In vitro*; SAR-CoV-2 M^pro^
**AutoGrow4**(35)	GA	Reaction-based mutation	*In silico* docking; poly(ADP-ribose) polymerase 1
**MoleGear**(40)	Graph-based EA	None	*In silico* docking/alignment; HIV-1 protease

### 3.1 Ligand Construction

A distinguishing aspect between methods is molecular representation. The structure of chemical entities resembles graphs in computer science, naturally leading to a molecular graph representation, where atoms and bonds correspond to nodes and edges. In contrast, the simplified molecular input line entry specification (SMILES) is a linear string representation that is derived from the molecular graphs.^41^ The difference in molecular representation is reflected in EA molecular construction operations: Graph-based methods alter a graph representation of the molecule,^39,40^ while SMILES-based methods modify a string representation.^35,37,38^ An advantage of using SMILES representation is rapid reaction-rule based modification through SMILES arbitrary target specification (SMARTS), as exemplified by Systemic Evolutionary Chemical Space Explorer (SECSE)^37^ and AutoGrow4.^35^

The initial population is often randomly drawn from drug-like libraries such as ZINC.^35,39^ Elend et al. trained a neural language model on a subset of the ZINC database to generate initial SMILES strings character by character.^38^ Dock_GA, SECSE, and MoleGear are fragment-based methods. Dock_GA generates a fragment library with the same infrastructure as *de novo* DOCK.^23,36^ SECSE proposed a fragment generation algorithm that can enumerate up to twelve heavy atom fragments and build up a collection containing 121 million fragments as the starting point of the workflow.^37^ MoleGear uses a fragment library generated from 1990 compounds selected from the National Cancer Institute diversity set.^40,42^

Structural operators vary slightly between the methods. Mutation is the most common operator that performs structural transformations like ring-open, ring-closing, atom insertion, or deletion, etc. In the Evolutionary Molecular Generation Algorithm (EMGA) proposed by Elend et al., the neural language model also serves as a mutation operator, and it randomly deletes, adds, and replaces atoms in a SMILES.^38^ AutoGrow4 has a reaction-rule based mutation operator using 36 click-chemistry reactions from AutoClickChem,^43^ 58 reactions published by Hartenfeller et al.,^44^ and any user-defined sets.^35^ In comparison, SECSE has both a classical mutation operator and a reaction operator. Elitism, as a feature of GA, is also common among the methods. SECSE introduced a graph-based deep learning module trained with docked samples of the population and can subsequently assess the quality of the rest of the population, speeding up the elite selection.^37^ The fragment-based methods also have a growing operator, similar to conventional fragment-based growing. Crossover is not as frequently employed, possibly due to its higher computational cost than other operators. Dock_GA has a 3D crossover operator that constructs molecules in the binding site environment.^36^ Besides the typical operators, SECSE also has a bioisostere operator that allows the interconversion of classical or nonclassical bioisosteric replacements.^37^

Fitness assessment for SB-CADD EA methods need to be fast since a large pool of molecules is proposed with every new population. Besides docking, similarity and diversity scores are also common fitness metrics. AutoGrow4 includes a diversity score that measures a molecule’s uniqueness relative to the others in the generation as an optional secondary metric.^35^ SECSE and MoleGear use similarity to reference known binders as the fitness metric in a mode parallel to the docking evaluation, therefore enabling ligand-based drug design as an option.^37,40^ SA metrics are also sometimes included in addition to the primary docking fitness score. SECSE includes a retrosynthesis module in its fitness evaluation,^37^ while Elend et al. and Steinmann et al. have an SA score component in their fitness functions.^38,39^ Other properties like drug-likeness are usually set as filters before docking,^35,37^ but there are exceptions such as Elend et al. which includes the drug-likeness and toxicity as weighted score terms in its fitness function.^38^

### 3.2 Synthetic Accessibility

Because of the nature of EA, it is harder to keep track of SA compared with other fragment-based growing or merging methods. The mutation and the crossover operators introduce complexity into the formation of new ligands, making reaction-rule based solutions hard to apply. Nevertheless, existing methods have incorporated reaction rules into the mutation operator^35^ or as a separate operator.^37^ Iterations of operations on a population also require the SA consideration to be fast since numerous molecules are assessed in the process. As a result, several approaches make use of SA scores as the solution.^38,39^ SA scores are metrics that measure the molecular complexity and are able to rank or filter large collections of molecules in a mere time.^45,46^ Retrosynthesis analysis is a more resource-intensive way to evaluate SA and is often utilized in postgeneration inspection.^37^ Although recent EA methods consider SA in one or more of the above directions, it is not uncommon to obtain ligands with poor SA scores or lengthy synthetic routes, as brought up in several methods. Top molecules proposed by SECSE, for example, were predicted to be synthesizable within 15 steps.^37^ Such concerns directly hinder the *in vitro* validation of the proposed molecules and limit the methods’ applicability.

### 3.3 Validation

Molecular docking was extensively used in the reviewed EA methods to report both the predicted binding pose and the predicted binding energy of the proposed molecules compared to a reference compound. The majority of the methods was able to propose molecules with similar predicted pose to the reference and better predicted binding energies. Steinmann et al. also compared the method’s performance to conventional high-throughput SBVS. All molecules in the ZINC subset where the initial population was sampled from were docked, and the top scores are compared to that of the generated molecules. For the case of DDR1 and β_2_AR, the reported methods found 1.9 times as many molecules with a good docking score (<−9.0) relative to known binders (−6.8/–6.9) by docking only 1.6 times as many molecules compared to SBVS.^39^ Besides docking, some methods also used MD simulation on a filtered list of top molecules to predict the binding energies, which is computationally more demanding.^38^

*In vitro* validation of the proposed molecules is generally lacking in these papers, most likely due to SA concerns. An early version of the method by Steinmann et al. was applied to SARS-CoV-2 M^pro^ in the COVID Moonshot project in 2020.^47^ One out of 10 submitted molecules proceeded to experimental validation but was later shown to have low inhibition.^39^

## 4 Monte Carlo Metropolis

Monte Carlo methods are a class of computational algorithms that solve problems through iterative random sampling. It has been extensively used in many optimization problems and sampling from probability distributions. The Metropolis criterion, which decides if the new state of each iteration is accepted or rejected, is often combined with the sampling. Monte Carlo Metropolis (MCM) methods are far from uncommon in CADD, with their utilization in fields ranging from molecular docking to small molecule drug discovery. For example, RosettaLigand has been a very successful example of applying MCM to flexible ligand docking.^48,49^ The employment of MC methods in *de novo* drug design can be traced back to 1991 in LEGEND,^10^ and the MCM concept has since been incorporated into the drug design workflows repeatedly during the 1990s.^50^ Yet at the time, most of these workflows adopted an atom-based ligand construction model, which suffered from exploding combinatorial search space concerns.^9,15^ For two decades, as fragment-based methods become more common and complement the shortcomings of early atom-based approaches, MCM methods have been silent in *de novo* drug design until recently.

In 2017, Oglic et al. designed a Metropolis-Hastings Markov Chain MC (MCMC) method which is then updated in the following year to perform active search in the chemical space.^15,51^ With a moderately active parent compound, candidates represented using vertex-labeled graphs are generated by substitutions at specific sites. The proposal generator includes various filters such as Lipinski’s rule and prohibition of specific synthetically inaccessible substitutions. The evaluation oracle performs rigid docking of a large number of ligand conformations. Binary feedback is returned based on a docking score threshold, and this feedback is incorporated in the Metropolis-Hastings criteria. The method was tested against integrin receptors that are important in idiopathic pulmonary fibrosis. A known inhibitor was used as the parent compound and, with constraints, defined the design space to around 185,000 compounds. The method was able to recover 19 out of 26 known actives with predicted binding affinity more favored than the parent compound. Although the result is encouraging, the SA considerations during molecular generation are weakly implemented, and the authors plan to incorporate actual reaction information into the algorithm in the future.

Xie et al. published an LB approach in 2021 that employs Metropolis-Hastings MCMC and graph neural network (GNN) to perform multiobjective drug discovery.^52^ Though being a novel attempt to combine conventional algorithms with machine learning tools, this approach named Markov Molecular Sampling is however beyond the discussion of the current review which focuses primarily on SB-CADD *de novo* design methods.

## 5 Machine Learning

Deep learning (DL) is a subclass of ML that incorporates multilayers of artificial neural networks (ANNs) to represent data in a rather complex latent space. Like other *de novo* design methods, DL methods need to solve the problems of molecular generation, property prediction, and molecular optimization, which are also the key differences between different DL methods.^53^ The success of deep generative models in other fields including natural language processing and computer vision has inspired the utilization of these sophisticated models in *de novo* drug design.

Molecular representation is an important aspect of DL *de novo* design methods, as it decides how a molecule is interpreted by the generative model. SMILES and graphs account for most of the two-dimensional (2D) representations used in the deep generative models in *de novo* drug design. Yet incorporating protein–ligand interactions with 2D representations is challenging, and years of efforts have mostly been LB, learning information primarily from known actives. Recently, several SB-CADD DL methods have been published to take advantage of the protein structures and build models trained on generic or target-specific databases to learn the intrinsic rules of protein–ligand interactions. Although some of these SB-CADD methods still employ the 2D representations with conformation generation processes to sample in the three-dimensional (3D) space, others adopt a 3D generative model where the configuration and conformation of a molecule are sampled simultaneously inside a protein pocket. These 3D generative models often need a 3D featurization for both the ligand and the protein. Such featurization includes cubic grid-based, Euclidean distance matrix (EDM)-based, and Cartesian coordinate-based, which have been reviewed in detail before.^16^

Over the past decade, there has been an increasing interest in DL methods, which can be seen by the soaring number of papers. There has been plenty of discussion on DL *de novo* drug design in recent reviews, both LB and SB.^9,16,53^ For this review, we focus specifically on the SB-CADD DL methods (Table 3).

**Table 3:** Recent Structure-Based Deep Learning *De Novo* Drug Design Approaches

Method	Ligand Representation	Molecular Generation	Validation (method; target)
**DiffSBDD**(54)	3D coordinates	Diffusion	*In silico* docking; 100 proteins from CrossDocked2020 and 130 complexes from Binding MOAD
**RELATION**(55)	3D property grids	VAE/AAE	*In silico* docking; protein kinase B alpha, CDK2
**Ragoza et al.**(56)	3D property grids	VAE	*In silico* docking; 10 random proteins from CrossDocked2020 Mutation study, Shikimate kinase
**DeepLigBuilder**(57)	3D coordinates	MPNN	*In silico* docking; SARS-CoV-2 M^pro^
**SBMolGen**(58)	SMILES	RNN	*In silico* FMO and MD; CDK2, EGFR, AA2AR, ADRB2
**MolAICal**(59)	SMILES/graphs	Sequence-based/GNN	*In silico* MD; glucagon receptor, SARS-CoV-2 M^pro^
**Xu et al.**(60)	SMILES	RNN	*In silico* docking; mitogen-activated protein kinase 14
**DEVELOP**(61)	Graphs	GNN, CNN	*In silico* docking; menin-MLL
**LiGANN**(62)	3D property grids	BicycleGAN	*In silico* docking; delta opioid 7TM receptor, CHK1, TNNI3K, and IRAK-4 kinase
**Armstrong et al.**(63)	Graphs	GCN, VAE	*In silico* docking; protein–ligand complexes from scPDB
**Grechishnikova etal.**(64)	SMILES	Transformer	*In silico* docking; Insulin-like growth factor 1 receptor, VEGFR2
**cMolGPT**(65)	SMILES	Transformer	*In silico* QSAR prediction; EGFR, HTR1A, S1PR1
**Luo et al.**(66)	3D coordinates	GNN	*In silico* Docking; 100 proteins from CrossDocked2020

### 5.1 Ligand Construction

**Figure 3:**
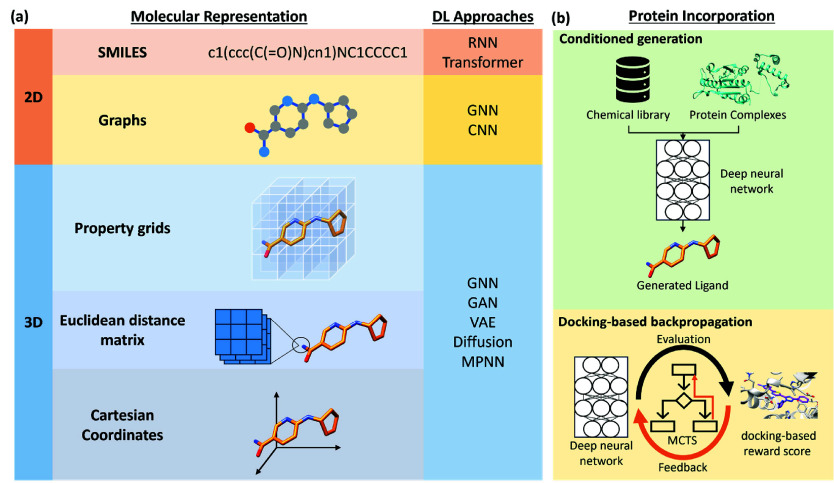
(a) Common molecular representations and deep learning models utilized in structure-based deep learning de novo design approaches. (b) Protein information can be included in ligand generation in two ways: conditioned generation and docking-based backpropagation.

The methods summarized here employ a variety of ANNs to generate ligands. The underlying difference is ligand representation (Figure 3a). SMILES-based methods generate strings character by character within a chemical context. Recurrent neural network (RNN), a sequence-based model commonly used in text generation tasks, serves this purpose well and for many years has been employed as the generative model in SMILES-based methods.^58,60^ More recently, transformer-based approaches have achieved success in many sequence processing tasks. The self-attention mechanism of transformer allows long-range dependencies, while also being faster than recurrent networks.^67^ Several groups have incorporated the transformer model into structure-based drug design.^64,65^ Graph-based methods represent atoms and bonds in nodes and edges, intuitively leading to the utilization of graph neural networks (GNNs) that process graph data.^59,61,63^ 3D-based methods are mostly cube grid-based, where the molecules are translated into property grids such as elements, aromaticity, hydrogen bond donors and acceptors, formal charge, etc. In these cases, a variational autoencoder (VAE)^55,56^ or a generative adversarial network (GAN)^62^ can perform the feature extraction and generate latent vectors that correspond to 3D druglike molecules. Then a decoder, usually a long short-term memory network, is required to transform these vectors into readable formats such as SMILES.^55,62^ For the same decoding problem, Ragoza et al. implemented an atom fitting algorithm that combines iterative atom detection with gradient descent to deduce a 3D molecular structure from a density grid.^56^ Besides grid-based featurization, other 3D-based methods adopt Cartesian coordinate-based representation, which generates rotationally and translationally invariant 3D embeddings that lead to full and unambiguous 3D structures.^54,57,66^ DiffSBDD implemented a diffusion model together with molecule inpainting to generate structures within the molecular context.^54^ DeepLigBuilder introduced a novel graph generative model, consisting of a state encoder with Message Passing Neural Network (MPNN) architecture and a policy network, to iteratively generate valid 3D druglike structures.^57^ Luo et al. used an autoregressive algorithm to sample atoms sequentially from a changing probability density, leading to unambiguous and multimodal ligand outputs.^66^

A significant difference between the methods is how the protein information is incorporated into ligand generation, as per the definition of SB. The methods reviewed here lie within one of the two categories: generation conditioned on target receptor and docking score backpropagation (Figure 3b). In the first type, the receptor is transformed into constraints or additional variables and integrated into the ligand generation model. How the receptor is represented or interpreted differs between methods, but a majority adopt a similar representation as the ligand representations mentioned above, and several methods encode the protein into constraint vectors via a convolution neural network (CNN).^56,60−62^ DiffSBDD used two strategies for protein conditioning: One considers the protein as a fixed 3D context during denoising of the diffusion model; the other learns the joint distribution of protein–ligand complexes.^54^ RELATION extracted pharmacophore features from crystal structures and the constraints were based on the root-mean-square of the matched feature pair distance and the number of matched pairs.^55^ Xu et al. transformed the binding site with a coarse-grained strategy using the sorted eigenvalues of the Coulomb matrix descriptor.^60^ Armstrong et al. represented the binding site in a graph-theoretic manner and trained their graph convolutional network (GCN) with EDM-based representation.^63^ The second type of method utilizes a common concept in DL called backpropagation to bias the ligand generation through a docking-based reward function. A Monte Carlo tree search (MCTS) can be combined into the molecular generation to optimize any intermediate state while guided by docking score, as done by DeepLigBuilder^57^ and SBMolGen.^58^ Apart from the two categories, MolAICal’s generative model involves no protein information and outputs fragments which then undergo classical SB-GA to generate complete ligands.^59^ The transformer proposed by Grechishnikova et al. considers the drug design problem as a translation from the amino acid sequence to SMILES and therefore needs only the protein sequence as the input.^64^

DL methods generate molecules through sampling in the latent space learned by the model. Unlike the real chemical space which is discrete, the latent space contains infinite points. DL generative models therefore can output an enormous number of molecules after training. These outputs need to be filtered and evaluated in a fast manner. Validity is a common metric to check in DL methods due to the continuous nature of the latent space, usually with cheminformatic toolkits like RDKit. Today most models can reach 80–90% validity by training on large chemical databases. Other filters and metrics include novelty, uniqueness, drug-likeness, and SA, as in conventional *de novo* design methods. Affinity prediction after molecular docking of the filtered molecules can be done with DL-based scoring functions trained with generic protein complex databases, as seen in several methods.^56,59,63^ To facilitate sampling and improve the quality of final outputs, RELATION employed Bayesian optimization with docking scores or quantitative structure–activity relationship (QSAR) scores and greatly improved the performance.^55^

### 5.2 Synthetic Accessibility

Despite the popularity, DL methods still face SA challenges. In fact, few DL methods consider SA in their protocol design and validation. It is somewhat understandable since layers of neural networks form a “black box” that makes it hard to incorporate SA measures. Some methods apply SA scores as a filter or metric to optimize during molecular generation.^58,63^ Most methods set the training data set to real molecules or drugs, expecting that SA can be a feature inherently learned. Another potential solution is multiobjective optimization. For most current deep generative models, predicted binding affinity is the primary and only objective to optimize during molecular generation. A multiobjective model would be able to optimize molecules based on several metrics other than predicted binding affinity, for example, drug-likeness and SA scores. Such multiobjective models have been realized by Armstrong et al. and also mentioned in the future work of several other methods.^55,58,63^

### 5.3 Validation

DL methods are black boxes. It is therefore important to test how each part of the model contributes to the overall performance during benchmarking. In SB-CADD *de novo* design, protein structural information is incorporated in many ways as discussed above. How much did the protein participate in molecular generation and did it bias the process as anticipated? This was investigated by several methods, especially those with a model conditioned on receptors. These methods built uncontrolled models that do not have access to protein information and compared the performance to the controlled models, usually by looking at recovery rate or similarity to known binders.^60,61^ Ragoza et al. answered these questions by evaluating the effect of mutation at the pocket on the outputs. For this purpose, shikimate kinase was mutated at interacting and noninteracting residues. The generative model responded to the pocket variants and generated molecules with corresponding changes.^56^ Methods that used docking-based MCTS molecular optimization compared the performance with and without MCTS.^57,58^ Some methods also looked at the effect of including known binders, either during training or as part of the input, where the generation process is much like lead optimization.^57,60^ Ragoza et al. designed a bias-toward-reference factor in their method that switches the mode between *de novo* design and lead optimization. In their benchmark, the latent space was interpolated by varying the bias factor during sampling, producing a series of ligands from novel ones to analogs.^56^

Further evaluation of DL methods proceeds analogously to conventional methods. In some cases where a 3D conformer can be directly generated, the quality of the generated pose can be compared with a docked pose. Ragoza et al., for example, compared the poses before and after minimization, and less than 20% of the generated molecules moved more than 2 Å RMSD, indicating that the majority of the molecules has a stable conformation in the pocket even before minimization.^56^ Pharmacophore recapture is also a common evaluation, especially for the cubic grid-based methods where properties are specifically encoded into molecular generation.^55,57,61^ Affinity prediction is typically done by molecular docking or MD simulation. The baseline performance is usually random decoys from the training database, and known binders are the next level of comparison. Most methods are able to generate molecules predicted to be as potent as known binders, and some are able to propose even better molecules. More than 15% of the generated molecules by Ragoza et al. have better predicted affinity than the reference molecules.^56^ SBMolGen was able to generate molecules with better predicted binding affinity than known actives for cyclin-dependent kinases 2 (CDK2), epidermal growth factor receptor erbB1 (EGFR), adenosine A2a receptor (AA2AR), and beta-2 adrenergic receptor (ADRB2).^58^ One-third of the molecules generated by DEVELOP and targeting menin and mixed lineage leukemia (MLL) fusion proteins have a predicted binding affinity greater or equal to the ground truth molecules.^61^ The method proposed by Luo et al. generated on average more than 29% of molecules with higher predicted affinity than reference ligands over 100 proteins from the CrossDock2020^68^ data set. Their method was also successful in linker prediction, recovering 48% of the test molecules.^66^

## 6 Conclusion

Since the rise of computer-based *de novo* design in the 1990s, methods in the field have evolved rapidly. *De novo* design has revolutionized drug discovery by developing *in silico* heuristics to speed up searching in the vast chemical space.^50^ The advantages of *de novo* design become more obvious when fragment-based libraries further speed up search. The relevance of SB-CADD *de novo* design is increasing as more and more crystal structures and homology models are available. The direct inclusion of target information in the search process makes the proposed molecules and their predicted interactions more exact and specific. As docking methods and computer power continue to improve, protein flexibility is not as obstructive as it once was, and new methods in the field all are able to address this. By providing novel scaffolds and constructive structural ideas, *de novo* design has aided medicinal chemists in developing patentable leads with desired properties.^19^ A great example of application is COVID-19, where *de novo* drug design methods made a rapid response to the newly discovered disease, yielding numerous novel drug molecules and reducing the time of development for treatment. This topic has been extensively reviewed elsewhere,^69^ and some of the methods that targeted COVID-19 are also included in this review.^36,38,39,57,59^

Despite the advances in the field, there are still challenges yet to be solved. Scoring remains a limiting factor for SB-CADD methods, with SB-CADD *de novo* design relying heavily on the performance of scoring functions. Conventional scoring methods behave poorly in screening, giving a low hit rate and many false positives.^17,70^ ML scoring functions are limited by the scope of their training sets, making scoring novel targets with few known binders unreliable.^18^ Quantitatively assessing the protein–ligand interactions in an accurate and fast manner is critical for the success of these methods. Iterative searching, scoring, and optimization in a vast library require a balance between accuracy and computing time. It can thus be foreseen that future advances in scoring functions will effectively improve the performance of SB-CADD *de novo* design methods.

SA is another common issue. Fragment-based methods try to overcome the problem by using druglike fragments and reaction-rule based molecular generation, but the SA diminishes during the process of iterative optimization. Other methods employ some sort of SA scores, sometimes as metrics to optimize and most times as filters. These scores measure the molecular complexity, usually calculated from a fixed set of molecules, and hardly agree with each other or the medicinal chemists. DL methods have even more concerns about SA, despite their popularity. The synthesizability of the training data set restricts the SA of the output molecules from the generative models, and SA is usually not an objective during training.^71^ This also calls for multiobjective optimization in the next stage of DL methods, where binding affinity is not the only metric to optimize, and more properties bias the molecular generation toward more druglike and synthetically accessible molecules.

From the reviewed methods, we also observed a lack of standardized benchmark workflow. A *de novo* design method should validate its proposed molecules experimentally, but such validation is rarely performed in these methods, partly because of the SA concerns mentioned above. Even *in silico*, there is no unified benchmark strategy in the field. Common evaluation strategies include structural and binding mode similarity to known binders, predicted binding affinities compared to known binders or random decoys, novelty and diversity (and validity for DL methods), and various drug-likeness metrics. The reviewed methods adopt one or more of these strategies, validating their proposed framework at different levels, making it difficult to compare the performance of different methods. With the rapid advance in the field and the emergence of numerous novel approaches, future methods should have more comprehensive benchmarks to convince the scientific public. Alternatively, public benchmarking exercises like Critical Assessment of Computational Hit-finding Experiments (CACHE) provide the community with opportunities to test the computational methods experimentally and under a standardized setting.^72^ Results from these exercises will be collected and released to the public, serving as valuable resources to guide further advancement. As the community grows with the abundance of available structures, we hope more SB-CADD *de novo* design methods can be adopted to facilitate future drug development.

Table 4 contains data and software availability.

**Table 4:** Data and Software Availability

Method	License	Source
**LigBuilder V3**(20)	Free for all	http://repharma.pku.edu.cn/ligbuilder3/
**NAOMInext**(21)	Free for academic	http://uhh.de/naomi
**PINGUI**(22)	Web application	www.kolblab.org/scubidoo/pingui
***de novo*DOCK**(23)	Free for academic	https://dock.compbio.ucsf.edu/
**AutoT&T 2**(24)	300 USD for academic;	http://www.sioc-ccbg.ac.cn/software/att2/
	3000 USD for industrial	
**OpenGrowth**(25)	Free for all	http://opengrowth.sourceforge.net/
**Frag4Lead**(27)	Not public	N/A
**LeadOp+R**(28)	Not public	N/A
**AutoCouple**(29)	Unknown	Scripts available at https://github.com/Caflisch-Group/AutoCouple_Python-based
**Dock_GA**(36)	Free for academic	https://dock.compbio.ucsf.edu/
**SECSE**(37)	Open source	https://github.com/KeenThera/SECSE
**EMGA**(38)	Not public	N/A
**Steinmann et al.**(39)	Open source	https://github.com/cstein/GB-GA/tree/feature-glide_docking
**AutoGrow4**(35)	Open source	https://durrantlab.pitt.edu/autogrow4/
**MoleGear**(40)	Not public	N/A
**Oglic et al.**(15)	Not public	N/A
**DiffSBDD**(54)	Open source	https://github.com/arneschneuing/DiffSBDD
**RELATION**(55)	Unknown	https://github.com/micahwang/RELATION
**Ragoza et al.**(56)	Open source	https://github.com/mattragoza/liGAN
**DeepLigBuilder**(57)	Not public	N/A
**SBMolGen**(58)	Open source	https://github.com/clinfo/SBMolGen
**MolAICal**(59)	Free for academic	https://molaical.github.io/
**Xu et al.**(60)	Not public	N/A
**DEVELOP**(61)	Open source	https://github.com/oxpig/DEVELOP
**LiGANN**(62)	Web application	https://playmolecule.com/LiGANN/
**Armstrong et al.**(63)	Not public	N/A
**Grechishnikova et al.**(64)	Unknown	https://github.com/dariagrechishnikova/molecule_structure_generation
**cMolGPT**(65)	Unknown	https://github.com/VV123/cMolGPT
**Luo et al.**(66)	Open source	https://github.com/luost26/3D-Generative-SBDD

## Data Availability

The data and software availability information can be found in the table at the end of the text.

## Abbreviations

AA2AR: adenosine A2a receptor
AAE: adversarial autoencoder
ADRB2: beta-2 adrenergic receptor
ANNs: artificial neural networks
SB-CADD: structure-based computer-aided drug design
CDK2: cyclin-dependent kinases 2
CNNs: convolutional neural networks
DL: deep learning
EAs: evolutionary algorithms
EDM: Euclidean distance matrix
EGFR: epidermal growth factor receptor erbB1
FMO: fragment molecular orbital
GAs: genetic algorithms
GANs: generative adversarial networks
GCN: graph convolution network
GNN: graph neural network
LB: ligand-based
MCM: Monte Carlo Metropolis
MCMC: Markov Chain Monte Carlo
MCTS: Monte Carlo tree search
MD: molecular dynamics
ML: machine learning
MM/GBSA: molecular mechanics with generalized Born and surface area solvation
RMSD: root-mean-square deviation
RNNs: recurrent neural networks
SA: synthetic accessibility
SBVS: structure-based virtual screening
SMILES: simplified molecular input line entry specification
VAE: variational autoencoders
VEGFR2: vascular endothelial growth factor receptor 2
β_2_AR: β_2_-adrenergic receptor

## References

[ref1] Sliwoski G.; Kothiwale S.; Meiler J.; Lowe E. W. Computational Methods in Drug Discovery. Pharmacol. Rev. 2014, 66, 334–395. 10.1124/pr.112.007336. 24381236 PMC3880464

[ref2] Śledź P.; Caflisch A. Protein Structure-Based Drug Design: From Docking to Molecular Dynamics. Curr. Opin. Struct. Biol. 2018, 48, 93–102. 10.1016/j.sbi.2017.10.010. 29149726

[ref3] Bhunia S. S.; Saxena A. K. Efficiency of Homology Modeling Assisted Molecular Docking in G-Protein Coupled Receptors. Curr. Top. Med. Chem. 2021, 21, 269–294. 10.2174/1568026620666200908165250. 32901584

[ref4] Song Y.; Dimaio F.; Wang R. Y. R.; Kim D.; Miles C.; Brunette T.; Thompson J.; Baker D. High-Resolution Comparative Modeling with RosettaCM. Structure 2013, 21, 1735–1742. 10.1016/j.str.2013.08.005. 24035711 PMC3811137

[ref5] Jumper J.; Evans R.; Pritzel A.; Green T.; Figurnov M.; Ronneberger O.; Tunyasuvunakool K.; Bates R.; Žídek A.; Potapenko A.; Bridgland A.; Meyer C.; Kohl S. A. A.; Ballard A. J.; Cowie A.; Romera-Paredes B.; Nikolov S.; Jain R.; Adler J.; Back T.; Petersen S.; Reiman D.; Clancy E.; Zielinski M.; Steinegger M.; Pacholska M.; Berghammer T.; Silver D.; Vinyals O.; Senior A. W.; Kavukcuoglu K.; Kohli P.; Hassabis D. Applying and Improving AlphaFold at CASP14. Proteins 2021, 89, 1711–1721. 10.1002/prot.26257. 34599769 PMC9299164

[ref6] Varadi M.; Anyango S.; Deshpande M.; Nair S.; Natassia C.; Yordanova G.; Yuan D.; Stroe O.; Wood G.; Laydon A.; Zídek A.; Green T.; Tunyasuvunakool K.; Petersen S.; Jumper J.; Clancy E.; Green R.; Vora A.; Lutfi M.; Figurnov M.; Cowie A.; Hobbs N.; Kohli P.; Kleywegt G.; Birney E.; Hassabis D.; Velankar S. AlphaFold Protein Structure Database: Massively Expanding the Structural Coverage of Protein-Sequence Space with High-Accuracy Models. Nucleic Acids Res. 2022, 50, D439–D444. 10.1093/nar/gkab1061. 34791371 PMC8728224

[ref7] Lipinski C.; Hopkins A. Navigating Chemical Space for Biology and Medicine. Nature 2004, 432, 855–861. 10.1038/nature03193. 15602551

[ref8] Schneider P.; Schneider G. De Novo Design at the Edge of Chaos. J. Med. Chem. 2016, 59, 4077–4086. 10.1021/acs.jmedchem.5b01849. 26881908

[ref9] Mouchlis V. D.; Afantitis A.; Serra A.; Fratello M.; Papadiamantis A. G.; Aidinis V.; Lynch I.; Greco D.; Melagraki G. Advances in De Novo Drug Design: From Conventional to Machine Learning Methods. Int. J. Mol. Sci. 2021, 22, 1676 10.3390/ijms22041676. 33562347 PMC7915729

[ref10] Nishibata Y.; Itai A. Automatic Creation of Drug Candidate Structures Based on Receptor Structure. Starting Point for Artificial Lead Generation. Tetrahedron 1991, 47, 8985–8990. 10.1016/S0040-4020(01)86503-0.

[ref11] de Souza Neto L. R.; Moreira-Filho J. T.; Neves B. J.; Maidana R. L. B. R.; Guimarães A. C. R.; Furnham N.; Andrade C. H.; Silva F. P. In Silico Strategies to Support Fragment-to-Lead Optimization in Drug Discovery. Front. Chem. 2020, 8, 93 10.3389/fchem.2020.00093. 32133344 PMC7040036

[ref12] Bienstock R. J. Computational Methods for Fragment-Based Ligand Design: Growing and Linking. Methods Mol. Biol. 2015, 1289, 119–135. 10.1007/978-1-4939-2486-8_10. 25709037

[ref13] Devi R. V.; Sathya S. S.; Coumar M. S. Evolutionary Algorithms for de Novo Drug Design - A Survey. Appl. Soft Comput. 2015, 27, 543–552. 10.1016/j.asoc.2014.09.042.

[ref14] Le T. C.; Winkler D. A. A Bright Future for Evolutionary Methods in Drug Design. ChemMedChem 2015, 10, 1296–1300. 10.1002/cmdc.201500161. 26059362

[ref15] Oglic D.; Oatley S. A.; Macdonald S. J. F.; Mcinally T.; Garnett R.; Hirst J. D.; Gärtner T. Active Search for Computer-Aided Drug Design. Mol. Inform. 2018, 37, 1700130 10.1002/minf.201700130. 29388736

[ref16] Xie W.; Wang F.; Li Y.; Lai L.; Pei J. Advances and Challenges in de Novo Drug Design Using Three-Dimensional Deep Generative Models. J. Chem. Inf. Model. 2022, 62, 2269 10.1021/acs.jcim.2c00042. 35544331

[ref17] Su M.; Yang Q.; Du Y.; Feng G.; Liu Z.; Li Y.; Wang R. Comparative Assessment of Scoring Functions: The CASF-2016 Update. J. Chem. Inf. Model. 2019, 59, 895–913. 10.1021/acs.jcim.8b00545. 30481020

[ref18] Su M.; Feng G.; Liu Z.; Li Y.; Wang R. Tapping on the Black Box: How Is the Scoring Power of a Machine-Learning Scoring Function Dependent on the Training Set?. J. Chem. Inf. Model. 2020, 60, 1122–1136. 10.1021/acs.jcim.9b00714. 32085675

[ref19] Schneider G.; Clark D. E. Automated De Novo Drug Design: Are We Nearly There Yet?. Angew. Chem. 2019, 131, 10906–10917. 10.1002/ange.201814681. 30730601

[ref20] Yuan Y.; Pei J.; Lai L. LigBuilder V3: A Multi-Target de Novo Drug Design Approach. Front. Chem. 2020, 8, 142 10.3389/fchem.2020.00142. 32181242 PMC7059350

[ref21] Sommer K.; Flachsenberg F.; Rarey M. NAOMInext - Synthetically Feasible Fragment Growing in a Structure-Based Design Context. Eur. J. Med. Chem. 2019, 163, 747–762. 10.1016/j.ejmech.2018.11.075. 30576905

[ref22] Chevillard F.; Rimmer H.; Betti C.; Pardon E.; Ballet S.; Van Hilten N.; Steyaert J.; Diederich W. E.; Kolb P. Binding-Site Compatible Fragment Growing Applied to the Design of β 2 -Adrenergic Receptor Ligands. J. Med. Chem. 2018, 61, 1118–1129. 10.1021/acs.jmedchem.7b01558. 29364664

[ref23] Allen W. J.; Fochtman B. C.; Balius T. E.; Rizzo R. C. Customizable de Novo Design Strategies for DOCK: Application to HIVgp41 and Other Therapeutic Targets. J. Comput. Chem. 2017, 38, 2641–2663. 10.1002/jcc.25052. 28940386 PMC5659719

[ref24] Li Y.; Zhao Z.; Liu Z.; Su M.; Wang R. AutoT&T v.2: An Efficient and Versatile Tool for Lead Structure Generation and Optimization. J. Chem. Inf. Model. 2016, 56, 435–453. 10.1021/acs.jcim.5b00691. 26799148

[ref25] Chéron N.; Jasty N.; Shakhnovich E. I. OpenGrowth: An Automated and Rational Algorithm for Finding New Protein Ligands. J. Med. Chem. 2016, 59, 4171–4188. 10.1021/acs.jmedchem.5b00886. 26356253

[ref26] Chen J.; Liu N.; Huang Y.; Wang Y.; Sun Y.; Wu Q.; Li D.; Gao S.; Wang H. W.; Huang N.; Qi X.; Wang X. Structure of PDE3A-SLFN12 Complex and Structure-Based Design for a Potent Apoptosis Inducer of Tumor Cells. Nat. Commun. 2021, 12, 6204 10.1038/s41467-021-26546-8. 34707099 PMC8551160

[ref27] Metz A.; Wollenhaupt J.; Glöckner S.; Messini N.; Huber S.; Barthel T.; Merabet A.; Gerber H. D.; Heine A.; Klebe G.; Weiss M. S. Frag4Lead: Growing Crystallographic Fragment Hits by Catalog Using Fragment-Guided Template Docking. Acta Crystallogr. Sect. D Struct. Biol. 2021, 77, 1168–1182. 10.1107/S2059798321008196. 34473087 PMC8411975

[ref28] Lin F.-Y.; Esposito E. X.; Tseng Y. J. LeadOp+R: Structure-Based Lead Optimization With Synthetic Accessibility. Front. Pharmacol. 2018, 9, 96 10.3389/fphar.2018.00096. 29556192 PMC5845126

[ref29] Batiste L.; Unzue A.; Dolbois A.; Hassler F.; Wang X.; Deerain N.; Zhu J.; Spiliotopoulos D.; Nevado C.; Caflisch A. Chemical Space Expansion of Bromodomain Ligands Guided by in Silico Virtual Couplings (AutoCouple). ACS Cent. Sci. 2018, 4, 180–188. 10.1021/acscentsci.7b00401. 29532017 PMC5833004

[ref30] Kutchukian P. S.; Lou D.; Shakhnovich E. I. FOG: Fragment Optimized Growth Algorithm for the de Novo Generation of Molecule: Occupying Druglike Chemical Space. J. Chem. Inf. Model. 2009, 49, 1630–1642. 10.1021/ci9000458. 19527020

[ref31] Warr W. A. A Short Review of Chemical Reaction Database Systems, Computer-Aided Synthesis Design, Reaction Prediction and Synthetic Feasibility. Mol. Inform. 2014, 33, 469–476. 10.1002/minf.201400052. 27485985

[ref32] Grygorenko O. O.; Radchenko D. S.; Dziuba I.; Chuprina A.; Gubina K. E.; Moroz Y. S. Generating Multibillion Chemical Space of Readily Accessible Screening Compounds. iScience 2020, 23, 101681 10.1016/j.isci.2020.101681. 33145486 PMC7593547

[ref33] Shang E.; Yuan Y.; Chen X.; Liu Y.; Pei J.; Lai L. De Novo Design of Multitarget Ligands with an Iterative Fragment-Growing Strategy. J. Chem. Inf. Model. 2014, 54, 1235–1241. 10.1021/ci500021v. 24611712

[ref34] Galletly J. Evolutionary Algorithms in Theory and Practice. Kybernetes 1998, 27, 979–980. 10.1108/k.1998.27.8.979.4.

[ref35] Spiegel J. O.; Durrant J. D. AutoGrow4: An Open-Source Genetic Algorithm for de Novo Drug Design and Lead Optimization. J. Cheminform. 2020, 12, 25 10.1186/s13321-020-00429-4. 33431021 PMC7165399

[ref36] Prentis L. E.; Singleton C. D.; Bickel J. D.; Allen W. J.; Rizzo R. C. A Molecular Evolution Algorithm for Ligand Design in DOCK. J. Comput. Chem. 2022, 43, 1942–1963. 10.1002/jcc.26993. 36073674 PMC9623574

[ref37] Lu C.; Liu S.; Shi W.; Yu J.; Zhou Z.; Zhang X.; Lu X.; Cai F.; Xia N.; Wang Y. Systemic Evolutionary Chemical Space Exploration for Drug Discovery. J. Cheminform. 2022, 14, 19 10.1186/s13321-022-00598-4. 35365231 PMC8973791

[ref38] Elend L.; Jacobsen L.; Cofala T.; Prellberg J.; Teusch T.; Kramer O.; Solov’Yov I. A. Design of SARS-CoV-2 Main Protease Inhibitors Using Artificial Intelligence and Molecular Dynamic Simulations. Molecules 2022, 27, 4020 10.3390/molecules27134020. 35807268 PMC9268208

[ref39] Steinmann C.; Jensen J. H. Using a Genetic Algorithm to Find Molecules with Good Docking Scores. PeerJ Phys. Chem. 2021, 3, e18 10.7717/peerj-pchem.18.

[ref40] Chu Y.; He X. MoleGear: A Java-Based Platform for Evolutionary De Novo Molecular Design. Molecules 2019, 24, 1444 10.3390/molecules24071444. 30979097 PMC6479339

[ref41] Weininger D. SMILES, a Chemical Language and Information System: 1: Introduction to Methodology and Encoding Rules. J. Chem. Inf. Comput. Sci. 1988, 28, 31–36. 10.1021/ci00057a005.

[ref42] Holbeck S. L. Update on NCI in Vitro Drug Screen Utilities. Eur. J. Cancer 2004, 40, 785–793. 10.1016/j.ejca.2003.11.022. 15120034

[ref43] Durrant J. D.; McCammon J. A. AutoClickChem: Click Chemistry in Silico. PLoS Comput. Biol. 2012, 8, e1002397 10.1371/journal.pcbi.1002397. 22438795 PMC3305364

[ref44] Hartenfeller M.; Eberle M.; Meier P.; Nieto-Oberhuber C.; Altmann K. H.; Schneider G.; Jacoby E.; Renner S. A Collection of Robust Organic Synthesis Reactions for in Silico Molecule Design. J. Chem. Inf. Model. 2011, 51, 3093–3098. 10.1021/ci200379p. 22077721

[ref45] Ertl P.; Schuffenhauer A. Estimation of Synthetic Accessibility Score of Drug-like Molecules Based on Molecular Complexity and Fragment Contributions. J. Cheminform. 2009, 1, 8 10.1186/1758-2946-1-8. 20298526 PMC3225829

[ref46] Coley C. W.; Rogers L.; Green W. H.; Jensen K. F. SCScore: Synthetic Complexity Learned from a Reaction Corpus. J. Chem. Inf. Model. 2018, 58, 252–261. 10.1021/acs.jcim.7b00622. 29309147

[ref47] Chodera J.; Lee A. A.; London N.; von Delft F. Crowdsourcing Drug Discovery for Pandemics. Nat. Chem. 2020 127 2020, 12, 581–581. 10.1038/s41557-020-0496-2. 32555379

[ref48] Meiler J.; Baker D. ROSETTALIGAND: Protein-Small Molecule Docking with Full Side-chain Flexibility. Proteins Struct. Funct. Bioinforma. 2006, 65, 538–548. 10.1002/prot.21086. 16972285

[ref49] Lemmon G.; Meiler J. Rosetta Ligand Docking with Flexible XML Protocols. Methods Mol. Biol. 2012, 819, 143–155. 10.1007/978-1-61779-465-0_10. 22183535 PMC3749076

[ref50] Schneider G.; Fechner U. Computer-Based de Novo Design of Drug-like Molecules. Nat. Rev. Drug Discov. 2005, 4, 649–663. 10.1038/nrd1799. 16056391

[ref51] Oglic D.; Garnett R.; Gaertner T. Active Search in Intensionally Specified Structured Spaces. Proc. AAAI Conf. Artif. Intell. 2017, 31, 2443–2449. 10.1609/aaai.v31i1.10930.

[ref52] Xie Y.; Shi C.; Zhou H.; Yang Y.; Zhang W.; Yu Y.; Li L. MARS: Markov Molecular Sampling for Multi-Objective Drug Discovery. ICLR; 2021.

[ref53] Zhang Y. An In-Depth Summary of Recent Artificial Intelligence Applications in Drug Design. arXiv Preprint. arXiv:2110.05478. 2021. https://arxiv.org/abs/2110.05478 (accessed 2024-03-09).

[ref54] Schneuing A.; Du Y.; Harris C.; Jamasb A.; Igashov I.; Du W.; Blundell T.; Lió P.; Gomes C.; Welling M.; Bronstein M.; Correia B. Structure-Based Drug Design with Equivariant Diffusion Models. Machine Learning for Structural Biology Workshop, NeurIPS 2022; 2022. 10.1038/s43588-024-00737-x PMC11659159 39653846

[ref55] Wang M.; Hsieh C.-Y.; Wang J.; Wang D.; Weng G.; Shen C.; Yao X.; Bing Z.; Li H.; Cao D.; Hou T. RELATION: A Deep Generative Model for Structure-Based De Novo Drug Design. J. Med. Chem. 2022, 65, 9478–9492. 10.1021/acs.jmedchem.2c00732. 35713420

[ref56] Ragoza M.; Masuda T.; Koes D. R. Generating 3D Molecules Conditional on Receptor Binding Sites with Deep Generative Models. Chem. Sci. 2022, 13, 2701–2713. 10.1039/D1SC05976A. 35356675 PMC8890264

[ref57] Li Y.; Pei J.; Lai L. Structure-Based de Novo Drug Design Using 3D Deep Generative Models. Chem. Sci. 2021, 12, 13664 10.1039/D1SC04444C. 34760151 PMC8549794

[ref58] Ma B.; Terayama K.; Matsumoto S.; Isaka Y.; Sasakura Y.; Iwata H.; Araki M.; Okuno Y. Structure-Based de Novo Molecular Generator Combined with Artificial Intelligence and Docking Simulations. J. Chem. Inf. Model. 2021, 61, 3304–3313. 10.1021/acs.jcim.1c00679. 34242036

[ref59] Bai Q.; Tan S.; Xu T.; Liu H.; Huang J.; Yao X. MolAICal: A Soft Tool for 3D Drug Design of Protein Targets by Artificial Intelligence and Classical Algorithm. Brief. Bioinform. 2021, 22, bbaa161 10.1093/bib/bbaa161. 32778891 PMC7454275

[ref60] Xu M.; Ran T.; Chen H. De Novo Molecule Design through the Molecular Generative Model Conditioned by 3D Information of Protein Binding Sites. J. Chem. Inf. Model. 2021, 61, 3240–3254. 10.1021/acs.jcim.0c01494. 34197105

[ref61] Imrie F.; Hadfield T. E.; Bradley A. R.; Deane C. M. Deep Generative Design with 3D Pharmacophoric Constraints. Chem. Sci. 2021, 12, 14577–14589. 10.1039/D1SC02436A. 34881010 PMC8580048

[ref62] Skalic M.; Sabbadin D.; Sattarov B.; Sciabola S.; Fabritiis G. De. From Target to Drug: Generative Modeling for the Multimodal Structure-Based Ligand Design. Mol. Pharmaceutics 2019, 16, 4282–4291. 10.1021/acs.molpharmaceut.9b00634. 31437001

[ref63] Aumentado-Armstrong T. Latent Molecular Optimization for Targeted Therapeutic Design. arXiv Preprint. arXiv:1809.02032. 2018. https://arxiv.org/abs/1809.02032 (accessed 2024-03-09).

[ref64] Grechishnikova D. Transformer Neural Network for Protein-Specific de Novo Drug Generation as a Machine Translation Problem. Sci. Rep. 2021, 11, 321 10.1038/s41598-020-79682-4. 33432013 PMC7801439

[ref65] Wang Y.; Zhao H.; Sciabola S.; Wang W. CMolGPT: A Conditional Generative Pre-Trained Transformer for Target-Specific De Novo Molecular Generation. Molecules 2023, 28, 4430 10.3390/molecules28114430. 37298906 PMC10254772

[ref66] Luo S.; Research H.; Guan J.; Ma J.; Peng J. A 3D Generative Model for Structure-Based Drug Design. Adv. Neural Inf. Process. Syst. 2021, 34, 6229–6239.

[ref67] Vaswani A.; Shazeer N.; Parmar N.; Uszkoreit J.; Jones L.; Gomez A. N.; Kaiser L.; Polosukhin I. Attention Is All You Need. Adv. Neural Inf. Process. Syst. 2017, 2017-Decem, 5999–6009.

[ref68] Francoeur P. G.; Masuda T.; Sunseri J.; Jia A.; Iovanisci R. B.; Snyder I.; Koes D. R. Three-Dimensional Convolutional Neural Networks and a Crossdocked Data Set for Structure-Based Drug Design. J. Chem. Inf. Model. 2020, 60, 4200–4215. 10.1021/acs.jcim.0c00411. 32865404 PMC8902699

[ref69] Floresta G.; Zagni C.; Gentile D.; Patamia V.; Rescifina A. Artificial Intelligence Technologies for COVID-19 De Novo Drug Design. Int. J. Mol. Sci. 2022, Vol. 23, Page 3261 2022, 23, 3261 10.3390/ijms23063261. PMC8949797 35328682

[ref70] Smith S. T.; Meiler J. Assessing Multiple Score Functions in Rosetta for Drug Discovery. PLoS One 2020, 15, e0240450 10.1371/journal.pone.0240450. 33044994 PMC7549810

[ref71] Gao W.; Coley C. W. The Synthesizability of Molecules Proposed by Generative Models. J. Chem. Inf. Model. 2020, 60, 5714–5723. 10.1021/acs.jcim.0c00174. 32250616

[ref72] Ackloo S.; Al-awar R.; Amaro R. E.; Arrowsmith C. H.; Azevedo H.; Batey R. A.; Bengio Y.; Betz U. A. K.; Bologa C. G.; Chodera J. D.; Cornell W. D.; Dunham I.; Ecker G. F.; Edfeldt K.; Edwards A. M.; Gilson M. K.; Gordijo C. R.; Hessler G.; Hillisch A.; Hogner A.; Irwin J. J.; Jansen J. M.; Kuhn D.; Leach A. R.; Lee A. A.; Lessel U.; Morgan M. R.; Moult J.; Muegge I.; Oprea T. I.; Perry B. G.; Riley P.; Rousseaux S. A. L.; Saikatendu K. S.; Santhakumar V.; Schapira M.; Scholten C.; Todd M. H.; Vedadi M.; Volkamer A.; Willson T. M. CACHE (Critical Assessment of Computational Hit-Finding Experiments): A Public-Private Partnership Benchmarking Initiative to Enable the Development of Computational Methods for Hit-Finding. Nat. Rev. Chem. 2022, 6, 287 10.1038/s41570-022-00363-z. 35783295 PMC9246350

